# Enablers of *Candida auris* persistence on medical devices and their mode of eradication

**DOI:** 10.18502/CMM.2023.150673

**Published:** 2023-03

**Authors:** Kingsley Chukwuemeka Nwachukwu, Ebubechukwu Nwarunma, Chinaza David Uchenna, Ositadinma Chinyere Ugbogu

**Affiliations:** 1 Department of Microbiology, Faculty of Biological Sciences, Abia State University, Uturu, Nigeria; 2 Department of Biological and Biomedical Science, School of Health and Life Sciences, Glasgow Caledonian University, Scotland, UK

**Keywords:** Biofilm, *Candida auris*, Enablers, Medical devices, Persistence

## Abstract

*Candida auris* is an emerging pathogen predominantly isolated from immunocompromised patients, hospitalized for a long time. It inhabits the skin surfaces of patients causing ear, wound, and systemic infections; if not treated properly, it could lead to severe mortality. Apart from being a skin pathogen, *C. auris* colonizes the surfaces of medical devices. Medical devices are hospital tools and components often utilized for the diagnosis and treatment of diseases associated with human skin. The mechanism of survival and persistence of *C. auris* on medical devices has remained unclear and is a serious concern for clinicians. The persistence of *C. auris* on medical devices has deterred its effective elimination, hindered the treatment of infections, and increased its antifungal resistance. Evidence has shown that a few surface molecules on the cell wall of *C. auris* and the extracellular matrix of the biofilm are responsible for its persistence and exist as enablers. Due to the increased cases of ear, skin, and systemic infections as well as death resulting from the spread of *C. auris* in hospitals, there is a need to study these enablers. This review focused on the identification of the enablers and aimed to evaluate them in relation to their ability to induce persistence in *C. auris*. In order to reduce the spread of or completely eliminate *C. auris* and its enablers in hospitals, the efficacy of disinfection and sterilization methods were compared.

## Introduction

*Candida auris*, an emerging pathogen has been reported to cause life-threatening infections in hospitalized patients with severe death output due to the development of resistance to most antifungal drugs [ 1 ]. It is often isolated from skin surfaces, blood and urine of these hospitalized patients [ 2 , 3 ]. After the first reported case of *C. auris* in Japan, the pathogen has spread to other countries within and outside Asia [ 4 ]. South Africa, America and Europe are the major hits [ 5 ]. Before the isolation of the fifth Clade of *C. auris* in Iran, four Clades (I, II, III and IV) had been identified in South Asia, East Asia, South Africa and South America respectively [ 6 ]. Among the five clades, Clades I, III and IV are more severe and cause candidemia [ 7 ] while Clade II infest the ear canal [ 8 ]. Through contact with affected persons and contaminated surfaces of medical devices, the spread of *C. auris* became rapid and spanned over 30 countries on six continents [ 9 ]. Globally, there has not been any verifiable record of *C. auris* cases or death rate, however, on October 25, 2022, World Health Organization included *C. auris* among the four most dreaded fungal pathogens in its ‘fungal priority pathogens list’ alongside *Cryptococcus neoformans*, *Aspergillus fumigatus*, *Candida albicans* [ 10 ]. In the US for instance, between 2019 and 2021, 17 states were affected with a total of 4,041 cases screened. The astronomical rise from 476 in 2019 to 1,471 in 2021 is worrisome. The risk factors associated with *C. auris* are not different from other *Candida* species [ 11 ]. Older patients with low immunity, admitted to hospitals for a long time and regularly exposed to groups of antimicrobials not less than 90 days are at risk of *C. auris* infection [ 11 , 12 ]. 

Medical devices are equipment specially designed for the diagnosis and treatment of diseases in hospitals and are often associated with human skin. The contamination of medical devices and colonization of human skin promote the easy spread of *C. auris* [ 13 ]. Its ability to survive and persist for a long time on some medical devices, including catheters, has resulted in skin and bloodstream infections among hospitalized patients and hospital workers [ 12 , 14 ]. Biofilm and adherence protein formation in *C. auris* are responsible for the colonization of medical devices. Biofilm in *C. auris* develops through several adaptive mechanisms which are dependent on their clades of origin [ 11 ]. Due to the long-lasting nature of biofilm on these surfaces, *C. auris* tends to thrive and produce diseases that are perhaps detrimental to humans. 

Although the mechanism of *C. auris* persistence in medical devices is still unclear, this review focused on the identification and examination of some of the enablers in the cell wall and extracellular matrix of the biofilm that support the adherence and persistence of *C. auris*. Moreover, the efficacy of sterilization of medical devices was evaluated in the present study.

## Materials and Methods

The authors searched original articles in PubMed, Science Direct, and Springer. The articles extracted were reviewed and focused on the enablers of the persistence of *C. auris* on the skin and medical devices. The keywords used for the search include enablers of *Candida auris*, persistence of *Candida auris*, candidemia, medical devices. Other keyword used include virulence factors, epidemiology, risk factors, pathogenesis and treatment of *Candida auris*. The title and abstract of each article were properly read and downloaded before the screening. Articles written in English and recently published between 2015 and 2023 were used for this study. Original papers not written in English and lacking merit and irrelevant to the study were discarded.

### Candida auris

*C. auris* is an emerging fungus that colonizes and persists on the human host’s skin, causing ear, wound and bloodstream infections in hospitals. It can be isolated from blood, urine, ear and parts of the skin of infected patients [ 11 ]. It hardly survives in anaerobic regions of the gut and mucosa. Since the isolation of the first strain in the first reported case in Japan, other strains have conquered territories globally. Approximately 5 clades have been isolated between 2009 and till date in the majority of countries in Asia, America, and Africa. The strain isolated from a particular region had similar genetic characteristics but differs from strains outside that region. In a genomic study performed by Lockhart and colleagues [ 15 ], over 7 chromosomes were identified; with an estimated 5,500 genes between 12.1 to 12.7 Mb [ 16 ]. However, all the strains share similar phenotypic features. Identification of *C. auris* is a problem for clinicians. Except for CHROMagar which produced pink colonies [ 17 , 18 ], standard laboratory assay could be misleading due to its close resemblance with other *Candida* species. Even the pink colonies on the CHROMagar plate were never a confirmatory test for *C. auris* as *C. haemulonii* had similar coloured colonies [ 4 ]. To confirm the presence of *C. auris*, matrix-assisted laser desorption ionization-time of flight mass spectrometry (MALDI-TOF MS) and internal transcribed spacer (ITS) sequencing is often used which fortunately remains a gold standard [ 4 , 5 , 19 ]. Other than the molecular confirmation of *C. auris*, one distinguishing feature is its ability to survive under high salt and temperature conditions. It possesses phospholipase, proteinase, adherence, and biofilm, which are prominent virulence factors responsible for its pathogenicity [ 4 , 7 ]. Unlike *C. albicans*, it is haploid [ 20 ] and does not germinate hyphae [ 21 ]. Treatment of *C. auris* infections has been a serious problem as it is resistant to most frontline antifungals such as polyenes, azoles and echinocandins [ 18 ]. Despite the reported susceptibility of *C. auris* to only echinocandins, a death rate resulting from *C. auris* infection of about 40% has been recorded [ 20 ].

### Candida auris and medical device

A medical device is any apparatus, tool, equipment or software used for the detection, control, and spread of infectious diseases which could be used separately or combined with any agent or accessories for human health [ 22 ]. Medical devices are major sources of *Candida*-related diseases in immunocompromised patients and persons making use of catheters (Table 1) [ 23 ]. Medical devices might be used once (single-use devices) [ 24 ] or reused [ 25 ], thus making contamination by pathogens certain. These devices could be used in tertiary hospitals by well-trained health practitioners or at homes and rural hospitals by amateurs; they help health workers detect and treat infectious diseases [ 22 ]. Decontamination is necessary to eliminate or reduce the spread of pathogens [ 26 ]. World Health Organization grouped reusable medical devices into critical risk, semi-critical risk and non-critical risk based on the type and degree of decontamination needed for each device to maintain clinical safety. In their recommendation, critical risk medical devices require total decontamination or sterility, semi-critical risk needs only disinfection while washing or sanitization of devices is required in non-critical risk type [ 25 ]. 

**Table 1:** Different sources of Candida auris isolates and the period of persistence before elimination

Medical device sources	Biological sources	Fomite sources	Period of persistence (median time)	References
Skin-surface temperature probes	ND	ND	61 days	[ 38 ]
Central venous catheters	Nose, axilla, groin, throat, rectum, and vascular line	Bedside trollies, radiators, windowsills, keypads, and equipment monitors	More than 4 h	[ 57 ]
Deep venous catheters	Rectum and nose	ND	18 months	[ 58 ]
ND	Nose, throat, axilla, groin, perineum, rectum, urine	ND	12 months	[ 3 ]
Bed railing, bed sheets, pillow, bedside trollies, floor, and air conditioner	Ear, nose, axilla, groin	Intravenous pole and oxygen mask were colonized by *C. auris*, while thermometer, blood pressure cuffs, nebulizer, ECG clip and sucker, and wheelchair had no *C. auris*	6 months	[ 59 ]
ND	Blood	Tunneled catheter	ND	[ 60 ]
Tables, beds, floors, walls, keyboards, and screens	Blood, vascular line, rectum,	ND	10 months and 3 weeks	[ 61 ]
Plastics	Groin, urine, stool, vagina, rectum, axilla, nares	ND	14 days	[ 33 ]
ND	Intraperitoneal cavity (intraperitoneal infection model), kidney	Catheter (mice subcutaneous model)	7 days for intraperitoneal cavity and 3 days for catheter	[ 36 ]
ND	ND	Central venous catheter (mice subcutaneous model)	2 days	[ 47 ]
Polymer, cellulose matrix, and steel	ND	ND	2 days	[ 55 ]
Hospital clothes	ND	ND	3 days	[ 37 ]

Key: ND =Not determined

*Candida* species adhere to hospital devices [ 27 ] and develop biofilm on medical devices used *in vivo* [ 28 , 29 ], hence making it very impossible to be eliminated, even when strong chemical agents are used. The presence of biofilm on the surfaces of the prosthetic devices leads to candidemia [ 30 ]; which results especially in patients with underlying ailments or whose immunity has deteriorated [ 31 ] due to overdependence in antifungals. 

*C. auris* colonizes the surfaces of hospital facilities including medical devices [ 32 ]. This is because, they can survive in polyethene material [ 33 ], metal surfaces [ 34 ], or both materials [ 35 ] even in dry conditions. Through insertion into the skin layer, these medical devices serve as vehicles for the transmission of diseases [ 30 ]. As stated initially, the cell wall of *C. auris* provides umpteen mechanisms for survival on medical devices. Comparing biofilm formation between *C. auris* and *C. albicans* using the scanning electron microscope in a rat catheter model, Vila et al. [ 36 ] discovered that *C. auris* had more biofilm than *C. albicans* on the catheter as a result of extracellular matrix (ECM)of the biofilm, which enhances its attachment and persistence. 

### Candida auris and persistence

There are different views on the persistence of *C. auris* on medical devices and/or the skin of animals or humans. Some authors believed that the mechanism of such action is unknown [ 37 ], others attributed the persistence indirectly to several factors. Eyre et al. [ 38 ] suggested that the ability of favourable adaptation of the yeast to the environment aids in its persistence and propagation for a long time while Heaney et al. [ 37 ] had a contrary opinion. Heaney and colleagues reported that the persistence of *C. auris* on medical devices did not correlate with its adaptation of the environment. Mitchell et al. [ 39 ] and Al-Fattani & Douglas [ 40 ] suggested that it results from the extracellular matrix of its biofilm. 

In most immunocompromised patients, it is present not as normal flora, but, as a contaminant. Unlike other *Candida* spp., *C. auris* is not isolated from the enteric region of the human host, rather, they could be found in the skin or mucosal regions where they cause infections, should the integrity of the skin surface be tampered with [ 1 ]. Biofilms developed and survive in places that have folds of skin and areas not constantly exposed [ 1 ]. Direct contact with contaminated skin surfaces or the use of any reusable medical device on the skin as a means of correcting health anomalies provides *C. auris* and its biofilm the needed environment to thrive [ 41 ]. Therefore, the ability to colonize and persist on skin surfaces and medical devices depends entirely on biofilm development [ 23 , 41 ]. The extracellular matrix (ECM) in biofilm shields the yeast cell from external stress, such as disinfectants and antifungals. It is yet to be known what the biofilm of *C. auris* gains by attaching to medical devices, as it is considered that the biofilms derive nutrients from the skin surface while surviving the high salinity and temperature associated with it [ 33 , 42 ]. Although, the biofilm may not acquire any nutrient from medical devices, its ability to persist for a long time through starvation, adherence and impermeability [ 36 ] makes them a superbug.

### Enablers of C. auris persistence

The mechanisms that possibly encourage the long-time persistence of *C. auris* on medical devices and resistance to antifungal treatments are yet to be studied. However, the major contributors of *C. auris* persistence are surface molecules present on ECM of the biofilm or cell wall. ECM is rich in polysaccharides which the organisms rely on for structure, protection and of course, resistance to most antifungals [ 39 ]. From the study, some molecules such as beta glucans, adhesins and Ngr1p were discovered which we referred to as “enablers”. They enable *C. auris* to adhere and persist not only on medical devices, but also on skin layer.

### Glucan production

**Figure 1:**
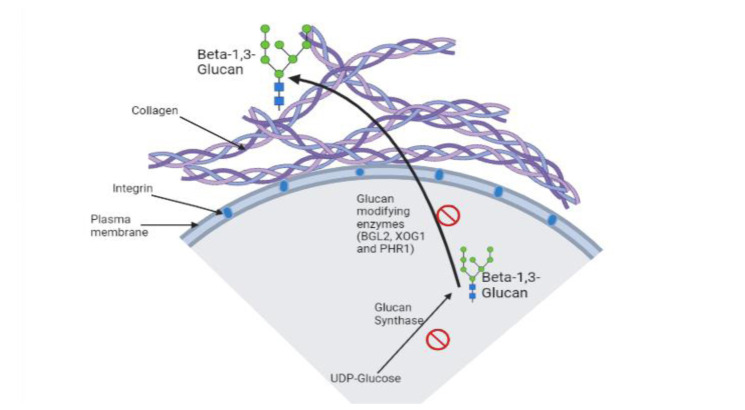
β-1,3-glucan delivery pathway, showing potential therapeutics target points

Generally, the cell wall of fungi is composed of chitin, mannans, glucans, proteins or their derivatives [ 43 ]. These cellular components are responsible for coordinating most metabolic, immunogenic [ 44 ], antifungal [ 39 ] and cellular permeable activities [ 45 ]. Each component has precedence over others based either on the nature of the fungi, that is, mould or yeast or cell wall dynamism [ 46 ]. For yeasts, their cell wall is mainly composed of beta-glucans; in fact, more than half of the dry weight of the entire yeasts is consistently built with glucans [ 46 ]. Each glucan is peculiar to species of *Candida* [ 47 ]. Glucans are made of (α, β)1,2, (α, β)1,3, (α, β)1,4 and (α, β)1,6 glucose linkage groups, however, the 1,3-glucose group is the most prominent of the other linkage units in *Candida* spp. β-1,3-D-glucan forms the motif through which other linkage groups attach. They are synthesized by glucan synthases, enzymes located inside the cell membrane through the encoding of *FKS1* and *FKS2* genes. Furthermore, glucan-altering enzymes play vital roles in delivering β-1,3-glucan to the extracellular matrix from the cells [ 48 ]. They further showed that these enzymes (BGL2, XOG1 and PHR1) are essential for the attachment of the biofilms to surfaces and the resistance of the biofilms to fluconazole antifungals [ 48 ]. Hence, glucan development, glucan delivery, and glucan alteration pathways can be considered effective therapeutic target points (Figure 1).

The cell walls apart from protecting to the yeast cells are also responsible for evading host immunological response, a mechanism that provides resistance to antifungal drugs in *C. auris*. When these structural motifs are destroyed, *C. auris* becomes susceptible to antifungals [ 49 ]. One contrasting factor among the *Candida* species is that the mechanism of immune evasion in *C. auris* differs from other Candida species [ 49 ]. The reason or mechanism for the persistence of *C. auris* in medical devices is yet to be discovered despite several attempts after its emergence in 2009. Some authors indirectly linked the enablers of persistence to biofilm and cell wall [ 38 ]. However, the components of the cell wall and the biofilm provide excellent evidence of *C. auris* persistence. Therefore, glucans, which are among the components of the cell wall, are delineated as one of the enablers of *C. auris* [ 47 ]. Furthermore, researchers argue that glucans could be a major contributor to the persistence of *C. auris* since they are among the compositions of extracellular matrix in *C. auris* biofilm and biofilm is linked to the survival of *C. auris* in the environment and medical devices and provides resistance to some antifungals [ 47 , 49 , 50 ].

### Adhesin proteins

**Figure 2:**
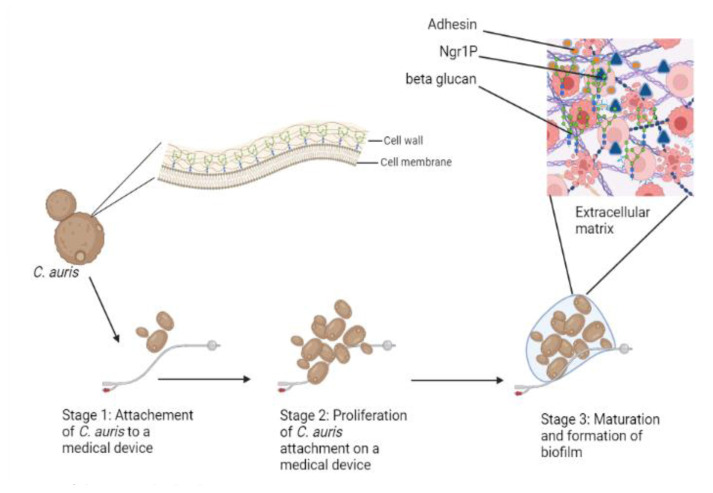
Step-wise processes of *C. auris* colonization and persistence on medical device (catheter) and its enablers in the extracellular matrix

Adhesins are a group of receptor proteins present in cell surfaces of microorganisms which promote interaction between a specific pathogen and the host. They are usually present in bacteria, mould and yeasts including *Candida* spp. Yeast cells generally use adhesins for effective attachment to their human host and adherence to medical devices through the formation of biofilms (Figure 2). They are also used for the enhancement of conjugation process among yeasts [ 51 ]. Although there are several adhesins already identified, each adhesin protein is peculiar to a particular yeast cell. For instance, among other molecules, *C. albicans* possesses agglutinin-like sequence (Als) groups; epithelial adhesion protein (EPA) is present in *C. glabrata* while *Saccharomyces cerevisiae* is known to have flocculins (FLO1, FLO5 and FLO9) [ 51 , 52 ]. Although the adhesins of *C. auris* are not yet clear, some researchers believe that since they share similar phylogeny with *C. albicans* and other *Candida* species, their genetic characteristics should differ appreciably [ 53 ]. *C. auris* possess Als3, a homologue of Als5 in *C. albicans*. In *C. auris*, Als3 protein was detected by *C. albicans* antibodies. Also, adhesin genes such as *ALS3* and *ALS4*, *ALS5* as well as *IFF4*, *CSA1*, *PGA52*, *PGA7*, *HYR3* were discovered to be present in a developed biofilm [ 54 , 55 ]. These genes are aggregate-forming in *C. auris* than in non-aggregate forming types [ 56 ]. 

They induce persistence in *C. auris* because they are responsible for biofilm development and confer resistance to antifungals [ 54 ]. For instance, Vila et al. [ 36 ], in an *in vivo* study suggested that attachment and persistence of biofilm on catheter was enabled by adhesin proteins of Als3 in *C. auris*; in the same vein, Kean et al. [ 55 ] in a transcriptomic study, concluded that *ALS1* and *ALS5* adhesin genes were responsible and enable adherence and persistence of biofilm of *C. auris* on plastics, steel metal, and paper.

### Ngr1proteins

Ngr1P is a zinc-finger domain molecule encoded by *NGR1* gene that represses the morphological expression of hyphae in *C. albicans* either by deletion of the gene or by overexpression [ 62 , 63 ]. Ngr1P is present in all *Candida* species, however, its functions differ. It regulates the ability to switch from yeast to hypha in some *Candida* species except in *C. auris*; it also regulates biofilm developmental stages in *C. albicans* [ 62 ]. However, both *C. albicans* and *C. auris* have NGR1 transcripts, a gene responsible for coding Ngr1p. In an experiment to determine the surface proteins of *C. auris*, performed by [ 64 ], it was discovered that both *C. auris* and *C. albicans* have similar Ngr1p protein components and are mainly located on the cell wall surfaces and ECM of the biofilm. Interestingly, Ngr1p was present in all the five clades (I, II, III, IV, V) of *C. auris* [ 11 ], which confirms the importance of this DNA-producing protein molecule. Sine *C. auris* does not synthesize a true hypha, the function of Ngr1p in that regard is yet to be ascertained [ 64 ]. It is known that biofilm development in C. auris enhances pathogenicity, thermotolerance, halotolerance, adhesion [ 22 ] and other virulence factors [ 32 ], therefore, since the Ngr1p regulates biofilm development, its presence possibly could be responsible for the attachment of *C. auris* on surfaces of skin and medical devices and further promote resistance to antifungals and recalcitrance to disinfection (Figure 2).

### The way forward: Is disinfection enough to prevent the persistence of C. auris on medical devices?

The use of disinfectants has been reported as an effective agent in eliminating *C. auris* persistence in the skin, medical devices and other surfaces [ 30 , 65 ]. However important this approach might seem to be; it is not enough to decontaminate surfaces. In most developing countries, due to the high cost of medical devices, lack of government policies and ignorance of the risks [ 26 ] health workers resorted to disinfecting the surfaces of single-use devices (SUD) contrary to WHO standards. Often, patients that utilize the SUD are usually unaware. Therefore, these devices should be used once and discarded immediately after use to prevent further spread of disease. In a 2022 report, for reusable devices, WHO recommended total sterilization of surgical and dental apparatus and other heat-stable devices which are critical and approved disinfection of surfaces with chemical agents for devices that do not penetrate the inner skin layer, that is, semi-critical devices. Some appropriate practices that can avert the spread of *C. auris* include proper use and management of medical devices in hospitals by health workers, total surveillance following the WHO recommended standard, and accurate application of transmission-based safeguards and sanitization of the hospital environment other than only the decontamination of medical devices only [ 13 ]. 

Hospital environment as well as medical devices have been implicated as major vehicles for the survival and persistence of *C. auris* [ 33 , 61 ]; thus, transmission of this yeast from the environment to hospitalized patients and staff is possible [ 66 ]. Moreover, through contact with the hospital facilities, health workers and infected patients deposit the pathogens from their bodies into the environment [ 2 , 57 ]. Therefore, attention should not only be on decontaminating medical devices or human skins [ 17 ], rather, the source of infection, which is the environment, should be the target [ 67 ]. Proper decontamination of the environment with appropriate measures will go along a way in eliminating the spread of *C. auris*.

## Conclusion

**Figure 3:**
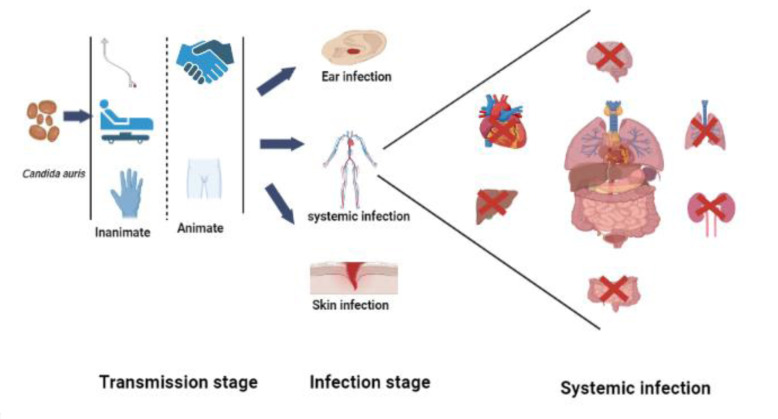
*Candida auris* contaminates both animate and inanimate surfaces, and contact with these surfaces produces a rapid spread of the pathogen and subsequently leads to infections. Between 30 to 90 days of infection, the pathogen spreads systemically while inhibiting the normal functions of vital organs and resulting in death

*Candida auris* infections can easily be transmitted by contact with contaminated animate and inanimate surfaces within the hospital environment (Figure 3). The enablers present on the cell wall and extracellular matrix of biofilm are glucans, adhesins, and Ngr1P. They contribute to a large extent to the persistence of *C. auris* on medical devices. The glucans protect the yeast from environmental stress while adhesins and Ngr1P control biofilm development and promote antifungal resistance. *C. auris* infections spread through skin contact with infected medical devices. To treat these infections, first we need to deal with the enablers present on the cell walls of *C. auris* and its biofilm. Going forward, instead of disinfecting medical devices only as often practiced in some hospitals, it will be most effective if total sterilization of medical surfaces is employed, as this is sustainable and will help reduce the spread of *C. auris*. 

## Acknowledgments

We thank Abia State University, Uturu We thank Abia State University, Uturu.

## Authors’ contribution

Conceptualization: K.C.N. Literature search: K.C.N., and C.D.U. Critical review and modification: O.C.U. Manuscript preparation: K.CN., O.C.U., and N.E. Illustrations: N.E., and K.C.N Proof reading: O.C.U and K.C.N. All authors provided critical revisions for important intellectual content and also read and approved the final manuscript.

## Conflicts of interest

We declare no conflict of interest.

## Financial disclosure

This research was not funded with any grant from any agency.
